# Severe atopic dermatitis treated with Dupilumab in a CTLA-4-deficient patient: A case report and review of the literature

**DOI:** 10.1177/2050313X241311042

**Published:** 2025-01-07

**Authors:** Ye-Jean Park, Jennifer Grossman, Lynne Robertson

**Affiliations:** 1 Temerty Faculty of Medicine, Division of Medicine, Department of Medicine, University of Toronto, Toronto, ON, Canada; 2 Division of Hematology, Department of Medicine, Cumming School of Medicine, University of Calgary, Calgary, AB, Canada; 3 Cumming School of Medicine, University of Calgary, Calgary, AB, Canada; 4 Division of Dermatology, Department of Medicine, Cumming School of Medicine, University of Calgary, Calgary, AB, Canada

**Keywords:** CTLA-4 deficiency, atopic dermatitis, Dupilumab

## Abstract

Atopic dermatitis is a chronic inflammatory skin disease associated with immune dysregulation, particularly overexpression of T helper 2 cytokines. Cytotoxic T lymphocyte-associated antigen 4 deficiency, a primary immune disorder, can exacerbate atopic dermatitis. Dupilumab, an IL-4 and IL-13 receptor antagonist, has demonstrated efficacy in controlling severe, recalcitrant atopic dermatitis by mitigating T helper 2-driven inflammation. We present a case of a 24-year-old male with cytotoxic T lymphocyte-associated antigen 4 haploinsufficiency and severe atopic dermatitis successfully managed with Dupilumab. The patient showed marked improvement in eczema severity scores, including a sixfold reduction in the Eczema Area and Severity Index and a threefold reduction in the Dermatology Life Quality Index over 6 months, highlighting Dupilumab’s potential role in cytotoxic T lymphocyte-associated antigen 4-deficient patients experiencing atopic dermatitis.

## Introduction

Atopic dermatitis (AD) is a chronic, prevalent inflammatory skin condition characterized by intense pruritus and a relapsing course.^1,2^ Its pathogenesis is closely linked to the dysregulation of the immune system, particularly the overexpression of T helper 2 (Th2) cells’ cytokines and chemokines.^3^ More specifically, interleukin-4 (IL-4) and interleukin-13 (IL-13) are produced by Th2 cells to induce the activation and differentiation of various immune cells, suppress the expression of proteins like filaggrin, and ultimately induce skin barrier dysfunction.^4^ The complex interplay of immune dysregulation and cutaneous manifestations of AD are highlighted in the primary immune disorder, cytotoxic T lymphocyte-associated antigen 4 (CTLA-4) deficiency (first reported in 2014).^5^ Given the numerous comorbidities associated with CTLA-4 deficiency, tailored therapeutic approaches and ongoing research endeavors are needed to optimize patient outcomes for AD occurring in this setting.

Dupilumab, a human monoclonal IgG4 antibody, has emerged as a promising treatment intervention for severe, recalcitrant AD.^2,6^ By competitively binding to the shared IL-4α subunit on the IL-4 and IL-13 receptors and upregulating proteins critical for epidermal barrier function, Dupilumab helps mitigate the Th2-driven inflammatory response.^7^

## Case report

**Figure 1.**
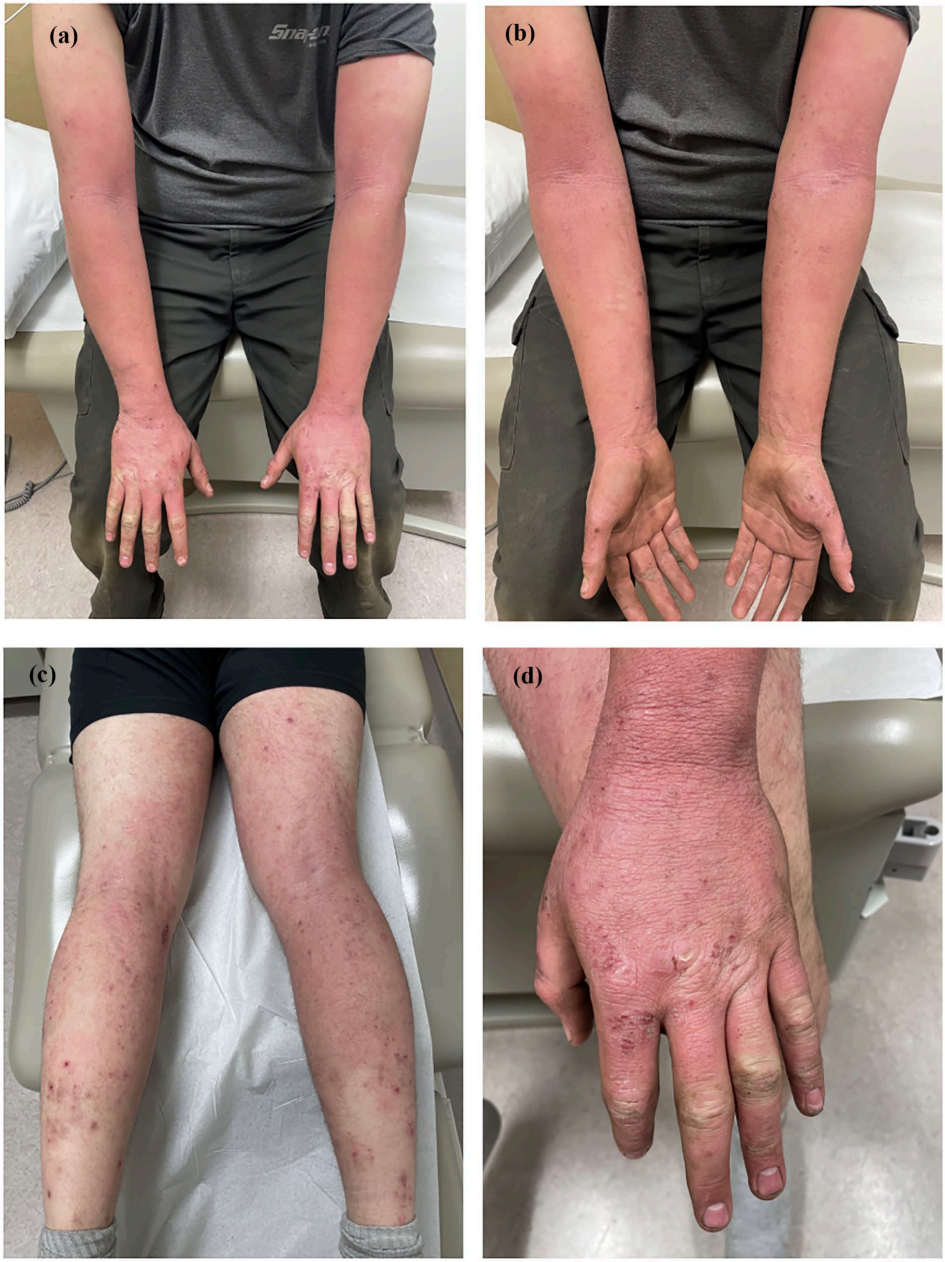
Clinical presentation of the patient with severe atopic dermatitis and CTLA-4 immunodeficiency prior to initiation of dupilumab (scores BSA 30%, IGA 4, EASI 32.4, and DLQI 25; a–d). The patient provided informed consent for the publication of these anonymized images and reports.

A 24-year-old male of Métis ethnicity presented to the clinic in January 2021 with chronic AD. This manifested as prominent generalized erythema and xerosis with superimposed papules and lichenified plaques across the extensor surfaces of the arms, the palms and dorsal hands, and the posterior aspects of the legs. The patient’s clinical evaluation included a body surface area (BSA) involvement of 30%, an investigator global assessment (IGA) score of 4, an Eczema Area and Severity Index (EASI) score of 32.4, and a Dermatology Life Quality Index (DLQI) score of 25 (Figure 1 ).

Regarding the patient’s medical history, he was diagnosed with AD in infancy, type 1 diabetes mellitus at age 3, and eosinophilic gastroenteritis complicated by eosinophilic ascites at age 21. Furthermore, the patient’s father had been diagnosed with and experienced symptoms of CTLA-4 haploinsufficiency 2 years earlier, including immune thrombocytopenia, severe AD, alopecia totalis, recurrent respiratory infections, and associated severe pulmonary hypertension. The patient’s father ultimately developed end-stage lung disease that resulted in terminal hypercarbic respiratory failure at the age of 50 years. Genetic testing revealed the patient also carried the pathogenic CTLA-4 mutation c.208C>T, p. R70W. The diagnosis of CTLA-4 haploinsufficiency was further corroborated by the patient’s predisposition to recurrent infections including a history of staphylococcal folliculitis and digital pyoderma, a perianal abscess, and several eye infections. There was no additional family history for CTLA-4 haploinsufficiency, recurrent infections, autoimmune endocrinopathies, enteropathies, cytopenias, or skin disorders.

To further assess the extent of the patient’s CTLA-4 immunodeficiency and its impact on the patient’s conditions, he underwent an immune profiling test. The patient had normal total numbers of T, B, and NK cells, though a slight reduction was noted in memory class-switched B cells. T-cell functionality was confirmed through appropriate proliferative responses to mitogen stimulation. Serologic testing revealed that protective titers against measles and mumps returned negative, while tetanus and diphtheria were maintained after a vaccine 5 years prior. Flow cytometry revealed a diminished expression of CD152 (also known as CTLA-4).

**Figure 2.**
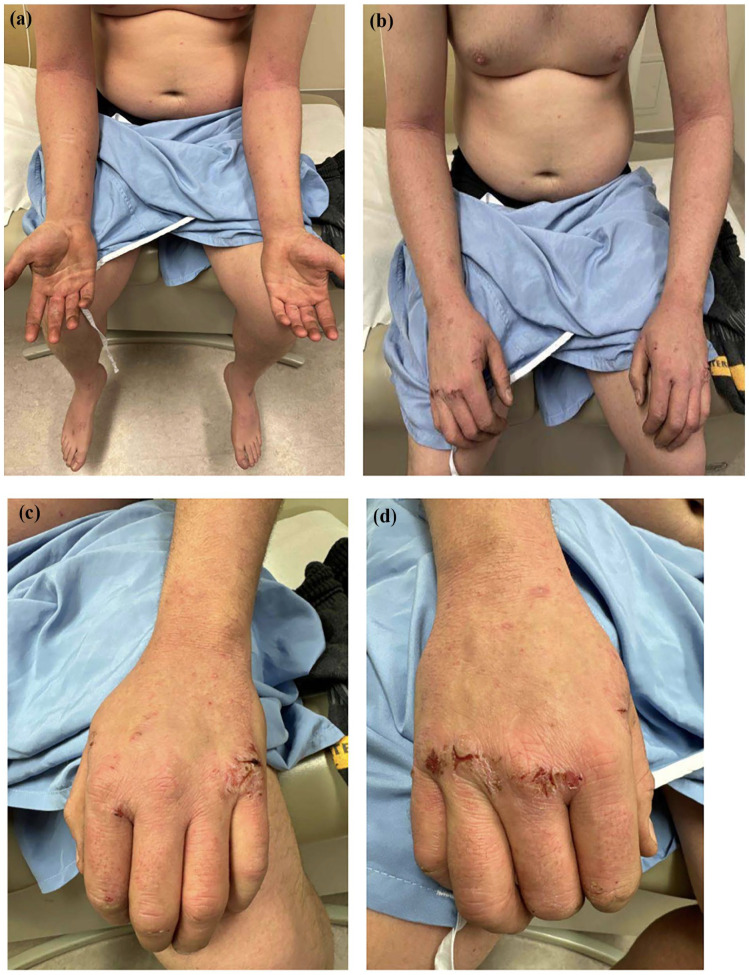
Clinical presentation of the patient with severe atopic dermatitis and CTLA-4 immunodeficiency 6 months after initiation of Dupilumab (scores BSA 6%, EASI 5.4 and DLQI 9). (a, b) Mild patchy eczema on forearms. (c, d) Improved eczema on hands with persistent eroded lichenified papules dorsal hands.

The patient was initially managed with betamethasone valerate 0.1% and clobetasol propionate 0.05% topical steroid ointments. His CTLA-4 haploinsufficiency was a relative contraindication to the use of traditional immunosuppressants such as methotrexate. In August 2023, the patient started Dupilumab, and within 6 months, his eczema severity scores improved dramatically with a sixfold reduction in the EASI (5.4), fivefold reduction in BSA (6%), and almost a threefold decrease in the DLQI (Figure 2). The patient was diagnosed with mild staphylococcal folliculitis on the arm while on Dupilumab but did not experience any side effects attributable to Dupilumab.

## Discussion

CTLA-4 functions by downregulating T-cell activation, thus preventing autoimmunity, hypogammaglobulinemia, and lymphoproliferation due to excess activated T-cell infiltration.^5,8,9^ Affected patients with a heterozygous loss-of-function mutation in CTLA-4 often present with a spectrum of clinical manifestations, including recurrent respiratory tract infections, cytopenia, endocrinopathies, gastrointestinal involvement, and notably, skin disorders like AD, psoriasis, and alopecia.^5,8^ Alongside de novo mutations, pathogenic variants from missense mutations, deletions, insertions, and nonsense mutations leading to CTLA-4 insufficiency pass down in an autosomal dominant fashion, as was the case for our patient. Abatacept, a humanized CTLA-4 fusion protein, may be used as a disease-specific treatment in CTLA-4-deficient patients; however, our patient had no other systemic indications for the initiation of this medication.^5^ Hematopoietic stem cell transplantation (HSCT) is the only curative treatment option available and is recommended for patients with refractory cytopenias, severe enteropathic disease, multi-organ involvement, or other features of severe disease.^5,9^

Dupilumab has emerged in the landscape of AD management in recent years and has been approved in Canada since 2017 for adult patients and since 2023 for pediatric patients with moderate-to-severe AD inadequately controlled with topical therapies.^10,11^ Through clinical trials and real-world data, Dupilumab has demonstrated considerable improvement in clinical outcomes with relatively uncommon side effects, including injection site reactions, nausea, and arthralgias.^12,13^

To our knowledge, this is one of the first case reports on the successful use of Dupilumab for severe AD associated with CTLA-4 deficiency. In one other report from Arruda et al. in 2023, a 20-year-old female patient with severe steroid-resistant AD and a different pathologic variant of CTLA-4 experienced significant improvement when treated with Dupilumab.^9^ Over 12 months, the patient’s EASI score decreased from 80.7 to 25.5 while her DLQI reduced from 27 to 1. The patient was concurrently managed with abatacept and intravenous immunoglobulin replacement for the CTLA-4 deficiency with no reported adverse effects.

In conclusion, dermatologists should consider CTLA-4 deficiency as a potential diagnosis in patients who present with symptoms of systemic immunodeficiency and severe concomitant skin diseases like AD.^14^ While no direct evidence links CTLA-4 deficiency to increased Dupilumab-related complications, underlying immunodeficiency may predispose patients to certain side effects associated with Dupilumab (including conjunctivitis, ulcerative keratitis, and eosinophilia), warranting careful monitoring.^15^ Nevertheless, when contrasted with systemic immunosuppressants such as methotrexate, corticosteroids, and Janus kinase inhibitors, Dupilumab may present a lower risk of opportunistic infections, malignancies, cytopenia, and enteropathies in patients with primary immunodeficiencies.^16^ Overall, Dupilumab is emerging as a promising option for managing severe AD in patients with CTLA-4 insufficiency.
